# Focal salvage treatment for radiorecurrent prostate cancer: A magnetic resonance-guided stereotactic body radiotherapy versus high-dose-rate brachytherapy planning study

**DOI:** 10.1016/j.phro.2020.07.006

**Published:** 2020-08-07

**Authors:** Thomas Willigenburg, Ellis Beld, Jochem Hes, Jan J.W. Lagendijk, Hans C.J. de Boer, Marinus A. Moerland, Jochem R.N. van der Voort van Zyp

**Affiliations:** University Medical Center Utrecht, Department of Radiation Oncology, 3508 GA Utrecht, The Netherlands

**Keywords:** Prostate cancer, MR-Linac, Focal salvage high-dose-rate brachytherapy, SBRT, Single fraction, MRI-guided radiotherapy, Radiotherapy treatment planning

## Abstract

**Background and Purpose:**

Magnetic resonance imaging (MRI)-guided focal salvage high-dose-rate brachytherapy (FS-HDR-BT) is one of the treatment options for radiorecurrent localized prostate cancer. However, due to the invasive nature of the treatment, not all patients are eligible. Magnetic resonance linear accelerator (MR-Linac) systems open up new treatment possibilities and could potentially replace FS-HDR-BT treatment. We conducted a planning study to investigate the feasibility of delivering a single 19 Gy dose to the recurrent lesion using a 1.5 Tesla MR-Linac system.

**Materials and Methods:**

Thirty patients who underwent FS-HDR-BT were included. The clinical target volume (CTV) encompassed the visible lesion plus a 5 mm margin. Treatment plans were created for a 1.5 Tesla MR-Linac system using a 1 mm planning target volume (PTV) margin. A dose of 19 Gy was prescribed to ≥ 95% of the PTV. In case this target could not be reached, i.e. when organs-at-risk (OAR) constraints were violated, a dose of ≥ 17 Gy to ≥ 90% of the PTV was accepted. MR-Linac plans were compared to clinical FS-HDR-BT plans.

**Results:**

Target dose coverage was achieved in 14/30 (47%) FS-HDR-BT plans and 17/30 (57%) MR-Linac plans, with comparable median D95% and D90%. In FS-HDR-BT plans, a larger volume reached ≥ 150% of the prescribed dose. Urethra D10%, rectum D1cm^3^, and rectum D2cm^3^ were lower in the FS-HDR-BT plans, while bladder dose was comparable for both modalities.

**Conclusion:**

Single fraction treatment of recurrent prostate cancer lesions may be feasible using stereotactic body radiotherapy (SBRT) on a MR-Linac system.

## Highlights

• SBRT may provide a non-invasive treatment option for recurrent prostate cancer.
• MR-Linac single fraction 19 Gy treatment of recurrent prostate cancer is feasible.
• MR-Linac dose distributions for the target were comparable to HDR-brachytherapy.
• Real-time intrafraction adaptation techniques are needed for clinical introduction.

## 1 Introduction

Up to 50% of the high-risk prostate cancer patients treated with primary dose-escalated radiation therapy develop a biochemical recurrence within 10 years of treatment [1], [2]. In more than half of the patients with a recurrence detected on imaging, the recurrence is confined to the prostate and/or seminal vesicles [3]. A treatment option for local radiorecurrent prostate cancer is magnetic resonance imaging (MRI)-guided focal salvage high-dose-rate brachytherapy (FS-HDR-BT) [4], [5]. With FS-HDR-BT, patients are treated in a single treatment session with a dose of 19 Gy to the clinical target volume (CTV). The radiation is delivered locally through catheters inserted via the perineum into the prostate using an Ir-192 source [4]. However, due to the invasiveness of the treatment and the need for spinal anesthesia, not all patients are eligible. Furthermore, a non-invasive treatment option will increase patient comfort.

The recent clinical introduction of magnetic resonance linear accelerator (MR-Linac) systems opens up a non-invasive treatment possibility that uses stereotactic body radiotherapy (SBRT) [6]. A MR-Linac integrates a MRI scanner with a linear accelerator [7]. This technology enables the visualization of the anatomy before and during treatment, allowing for daily adjustment of treatment plans [8], [9]. This leads to improved targeting compared to current conventional SBRT. Consequently, radiation-oncologists can reduce safety margins and deliver a higher dose per fraction. These aspects could make the MR-Linac a viable non-invasive alternative to FS-HDR-BT for treatment of radiorecurrent prostate cancer in a single fraction.

We conducted a planning study to evaluate the feasibility of delivering a single 19 Gy dose to a locally radiorecurrent prostate cancer lesion using a 1.5 Tesla MR-Linac system. The MR-Linac plans were compared to the clinically delivered FS-HDR-BT plans.

## 2 Materials and methods

### 2.1 Patient characteristics

**Fig. 1:**
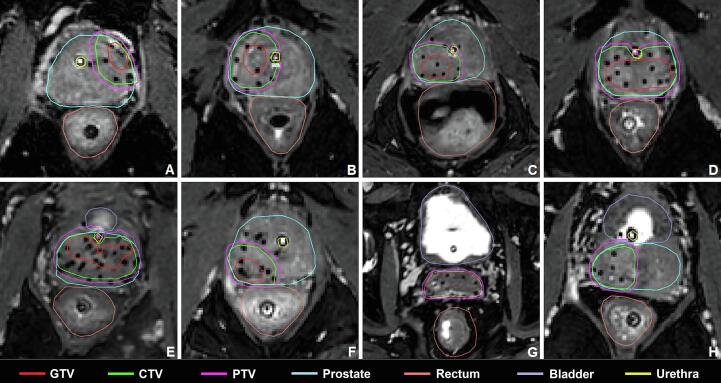
Different tumor locations treated with FS-HDR-BT. A: peripheral tumor, not near any of the organs-at-risk. B: central tumor adjacent to the urethra. C: peripheral/lateral tumor between rectum and urethra. D: central tumor between rectum and urethra. E: tumor adjacent to bladder and urethra. F: peripheral tumor adjacent to rectum and not near urethra. G: tumor in seminal vesicles, adjacent to rectum and/or bladder. H: tumor in base of the prostate, adjacent to rectum and bladder, without seminal vesicle involvement.

For this study, we included 30 patients that had previously been treated with FS-HDR-BT at the Department of Radiation Oncology of the UMC Utrecht between November 2014 and April 2019. Patients were included from two pre-existing FS-HDR-BT studies (Netherlands Trial Register numbers NTR6123 and NTR7014) approved by our institutional review board. Written informed consent was obtained for use of the data for this planning study. To make the results more generalizable, different tumor locations within the prostate were identified and patients were divided accordingly. Next, the 30 patients were selected randomly (using a random number table) from our patient database, taking into account the general tumor location, but without knowledge of the exact anatomy and FS-HDR-BT dose distribution. Baseline tumor characteristics of these 30 patients are displayed in Supplementary Table S1. The different tumor locations are visualized in Fig. 1.

### 2.2 Target definition

Pre-treatment delineations for the salvage FS-HDR-BT were performed using the in-house developed software Volumetool® [10] by experienced prostate cancer radiation oncologists on diagnostic MRI and prostate specific membrane antigen (PSMA) positron emission tomography (PET) images. The gross tumor volume (GTV) consisted of the visible tumor and was expanded uniformly by 5 mm to create the clinical target volume (CTV), staying inside the prostate body and/or seminal vesicle anatomical boundaries. Prostatic urethra, rectum, and bladder were delineated. For FS-HDR-BT treatment planning, the planning target volume (PTV) was equal to the CTV (i.e. a CTV to PTV margin of 0 mm). For MR-Linac treatment planning, a CTV to PTV margin of 1 mm was adopted. This 1 mm margin has to account for additional uncertainties that are introduced with the use of a MR-Linac system compared to FS-HDR-BT. These uncertainties consist of intrafraction prostatic motion and geometrical (in)accuracy of the system. Prior investigations by our department showed that the geometrical accuracy of the Elekta MR-Linac system is on average 0.3 mm (range 0.2–0.4 mm) [6]. Concerning prostate motion, we have developed an accurate soft tissue prostate tracking algorithm with a mean error of 0.07 mm (SD 0.22 mm) [11], [12]. Combined with new treatment planning systems developed at our department that facilitate online (intrafraction) replanning to account for anatomy shifts and changes, we assumed that a 1 mm PTV margin is achievable [9], [13], [14].

### 2.3 FS-HDR-BT treatment plan acquisition

The clinical FS-HDR-BT treatment plans, as delivered during treatment, were used in this study. During FS-HDR-BT, after insertion of the catheters, an intraoperative MRI scan was acquired. The diagnostic MRI scan was rigidly registered to the intraoperative MRI, followed by a transfer of the pre-treatment delineations to the intraoperative MRI. The delineations were adjusted according to the current anatomy. Furthermore, the positions of the brachytherapy catheters were reconstructed on the intraoperative MRI. The adjusted contours and reconstructed catheter positions were used for MRI-only dose calculation using Oncentra® Prostate (Version 4.2.2.4., Elekta). Between 6 and 15 catheters were used in these patients. A dose of at least 19 Gy was prescribed to 95% of the CTV (i.e. D95% ≥19 Gy). The dose constraints used for the organs-at-risk (OAR) are provided in Table 1. If the OAR constraints could not be met, a dose of at least 17 Gy to 90% of the CTV was accepted. The MRI-guided FS-HDR-BT treatment is extensively described elsewhere [4].

**Table 1:** Dose prescription and organs-at-risk constraints. *CTV for FS-HDR-BT plan and PTV for MR-Linac plan.

Target/OAR	Dosimetric parameter	Prescribed dose *or* constraint
CTV/PTV*	D95% D90%	≥ 19.0 Gy ≥ 17.0 Gy
*Urethra*	D10%	< 17.7 Gy
*Rectum*	D1cm^3^	< 12.0 Gy
*Bladder*	D1cm^3^	< 12.0 Gy

### 2.4 MR-Linac treatment plan acquisition

SBRT treatment planning requires patient body contours, which were missing for the FS-HDR-BT intraoperative MRI because of the small field-of-view. Therefore, the intraoperative MRI images were rigidly registered to preoperative computed tomography (CT) scans from diagnostic PET-CT examinations, using in-house developed software (Volumetool® [10]), which applies the normalized mutual information for registration. The contours were transferred to the CT scan. In case of a CT slice thickness of > 1 mm, the CT data set was resampled to a slice thickness of 1 mm using trilinear interpolation, allowing for correct contour propagation. The CT images with body contours and intraoperative FS-HDR-BT delineations were used for treatment planning. Treatment plans were created for an Elekta Unity MR-Linac, which integrates a 1.5 Tesla MRI scanner with a 7 MV linac mounted on a ring gantry, using Monaco (Version 5.40.01, 2019, Elekta). The planning software includes the beam characteristics of the MR-Linac and the presence of a magnetic field and cryostat. The beam characteristics have extensively been described by Woodings et al. [15]. Intensity-modulated radiotherapy (IMRT) plans were created using a 9-beam setup, with a 7 mm leaf width and a fixed collimator angle. The minimum segment width and area were 0.5 cm and 1.5 cm^2^, respectively. The minimum number of monitor units per segment was 5, with a maximum of 150 segments. A calculation grid spacing of 3 mm was used with a statistical uncertainty of 3% per control point and < 1% per voxel. A CTV to PTV margin of 1 mm was applied. The dose constraints were equal to those clinically applied in FS-HDR-BT. Hence, a dose of at least 19 Gy was prescribed to 95% of the PTV, allowing the CTV dose to reach above 19 Gy. OAR dose constraints are provided in Table 1. Furthermore, in case the OAR constraints could not be met, a dose of at least 17 Gy to 90% of the PTV was aimed for. Treatment planning was optimized for adequate target coverage with minimal dose to the OAR.

### 2.5 Evaluation of treatment plans

The simulated MR-Linac treatment plans were compared to the clinically delivered FS-HDR-BT plans. The CTV dose in the FS-HDR-BT plans was compared to the PTV dose in the MR-Linac plans. For the CTV/PTV target, the following dosimetric parameters were acquired: D90% and D95% (minimum dose to 90% and 95% of the target, respectively), V100%, V150%, and V200% (relative target volume receiving 100%, 150%, and 200% of the prescribed dose, respectively). For the bladder and rectum, D1cm^3^ and D2cm^3^ (highest dose to 1 cm^3^ and 2 cm^3^ of the OAR) were obtained. For the urethra, D10% (highest dose to 10% of the urethra) was collected. The median values (and range) of target volume coverage, high dose volumes and OAR doses were obtained. The paired data was compared using the non-parametric Wilcoxon signed-rank test. To correct for multiple testing, p-values < 0.01 were considered statistically significant. All analyses were performed using IBM SPSS Statistics 25 (Chicago, IL, USA).

## 3 Results

**Fig. 2:**
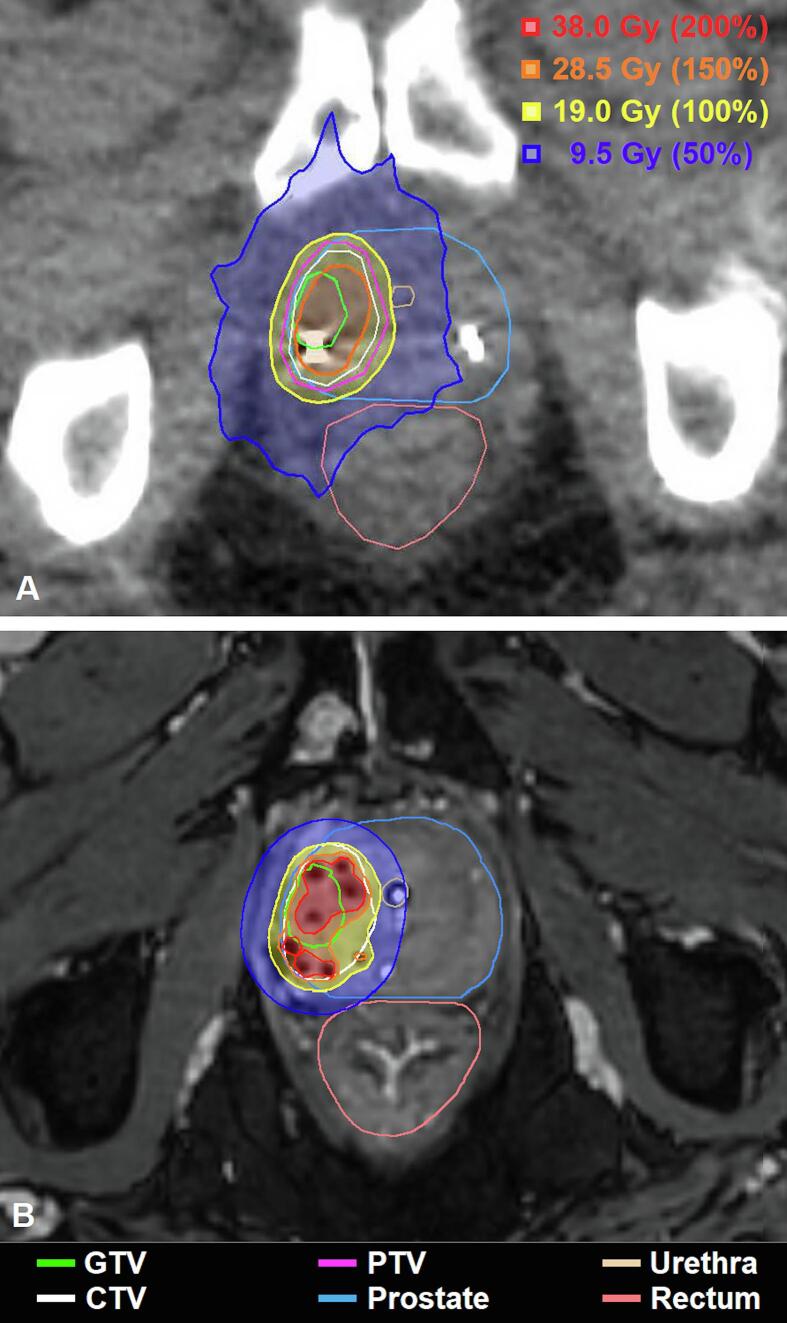
Example of dose distributions (with 50%, 100%, 150%, and 200% isodose-lines) in simulated MR-Linac plan (A) and clinically delivered FS-HDR-BT plan (B).

The delineated tumor volume (GTV) ranged from 0.7 cm^3^ to 17.8 cm^3^ and CTV volume ranged from 2.9 cm^3^ to 24.7 cm^3^ (Supplementary Table S1). Fig. 2 shows an exemplary dose distribution with the 50%, 100%, 150%, and 200% isodose-lines for a FS-HDR-BT and a MR-Linac plan.

Table 2 presents the dosimetric results for the FS-HDR-BT and MR-Linac plans. Out of the 30 FS-HDR-BT plans, 16 (53%) did not reach the prescribed dose of 19 Gy to ≥ 95% of the target, compared to 13 (43%) for the MR-Linac plans. Of the 16 plans that failed to meet the 19 Gy dose prescription for FS-HDR-BT, 5 succeeded with the MR-Linac. Contrarily, 2 out of the 13 that failed with the MR-Linac system, succeeded with FS-HDR-BT. Additionally, for both FS-HDR-BT and MR-Linac, two plans (7%) failed to meet the secondary dose prescription of at least 17 Gy to 90% of the target volume. All of these plans did, however, reach a dose of at least 17 Gy with the other treatment modality. The two plans that failed with FS-HDR-BT had a large CTV (33% and 60% of the prostate volume) surrounding the urethra. The two plans that failed with MR-Linac had a tumor located in the base and seminal vesicle, closely related to the bladder and rectum. In addition, no target volume received 200% (38.0 Gy) of the prescribed dose with MR-Linac treatment planning, whereas the median target volume receiving 38.0 Gy was 25% for FS-HDR-BT (p < 0.001). OAR dose constraints were met in all MR-Linac plans. In 5 (17%) FS-HDR-BT plans, OAR constraints were violated; in 3 (10%) cases the urethra D10% was > 17.7 Gy and in 2 cases (7%) the bladder D1cm^3^ was > 12 Gy. Median CTV/PTV D95% stratified by tumor location is presented in Table 3.

**Table 2:** Dosimetric parameters for FS-HDR-BT plans and MR-Linac plans for a single, 19 Gy dose prescription to the CTV and PTV respectively. *CTV for FS-HDR-BT and PTV for MR-Linac.

		FS-HDR-BT		MR-Linac		
Parameter	Reference dose	Median (n = 30)	Range	Median (n = 30)	Range	p-value
*Target coverage*						
D95% CTV/PTV*	≥19 Gy	18.8 Gy	14.0–21.7 Gy	19.0 Gy	15.5–21.7 Gy	0.053
D90% CTV/PTV*	≥17 Gy	20.3 Gy	15.9–23.8 Gy	20.2 Gy	16.7–22.6 Gy	0.484
*High dose volumes*						
V100% CTV/PTV*	–	95%	73–99%	95%	69–100%	0.894
V150% CTV/PTV*	–	55%	25–77%	1%	0–38%	<0.001
V200% CTV/PTV*	–	25%	9–54%	0%	0–0%	<0.001
*OAR dose*						
Urethra D10%	<17.7 Gy	16.0 Gy	6.7–18.2 Gy	17.5 Gy	8.2–17.7 Gy	<0.001
Rectum D1cm^3^	<12 Gy	10.2 Gy	4.1–12.0 Gy	12.0 Gy	9.4–12.0 Gy	<0.001
Rectum D2cm^3^	–	8.6 Gy	1.6–10.8 Gy	10.7 Gy	8.4–11.3 Gy	<0.001
Bladder D1cm^3^	<12 Gy	8.5 Gy	1.8–12.1 Gy	8.4 Gy	0.4–12.0 Gy	0.102
Bladder D2cm^3^	–	7.0 Gy	1.6–10.8 Gy	6.6 Gy	0.5–11.1 Gy	0.090

**Table 3:** Median D95% coverage with ranges for the different tumor locations (both anatomical location and position with respect to organs-at-risk) for FS-HDR-BT and MR-Linac plans respectively. U = urethra. R = rectum. B = bladder.

			FS-HDR-BT		MR-Linac	
Location	Near or between OAR?	N	Median D95 CTV (Gy)	Range (Gy)	Median D95 PTV (Gy)	Range (Gy)
*Peripheral*	No	5	20.0	19.1–21.1	21.0	20.4–22.0
*Central*	U	3	19.7	16.6–21.2	19.0	19.0–19.7
*Peripheral*	U and R	3	19.1	17.5–19.1	19.0	18.1–19.2
*Central*	U and R	4	18.4	17.7–18.7	18.5	17.4–19.3
*Central*	U and B	3	15.2	14.0–21.7	17.7	17.0–20.7
*Peripheral*	R	4	19.7	18.6–21.1	19.1	18.8–20.1
*Seminal vesicles*	R and/or B	5	16.8	16.0–19.2	17.9	15.5–19.1
*Base*	R and B	3	16.3	15.7–18.7	16.1	16.0–18.6

## 4 Discussion

We performed a comparative planning study to evaluate the feasibility of delivering a single 19 Gy dose to a local recurrent prostate cancer lesion using a 1.5 Tesla MR-Linac system. The simulated MR-Linac plans were compared to clinically delivered FS-HDR-BT plans. Both treatment planning techniques showed comparable target coverage (see Table 2), though with both techniques the target dose of 19 Gy was not reached for some cases. The SBRT technique combined with the additional 1 mm PTV margin resulted in a higher median dose to both the urethra and rectum for the MR-Linac plans. However, no OAR constraints were violated. Delivering a single 19 Gy dose to a recurrent prostate cancer lesion with acceptable target dose coverage, while respecting OAR constraints, thus seems feasible on a MR-Linac.

To our knowledge, no studies have investigated the feasibility of focal salvage SBRT to deliver a single fraction 19 Gy dose to an intraprostatic lesion in the recurrent setting with corresponding OAR constraints. Henderson et al. reported a planning study on single session treatment using SBRT and a 3 mm PTV margin [16]. They prescribed 15 Gy and 19 Gy to ≥ 95% and 65–75% of the PTV, respectively, with a boost of 21 Gy to ≥ 95% of the MRI-visible intraprostatic lesion plus a 3 mm margin. The median rectal D2cm^3^ was 14.4 Gy and median urethral V20.8 was 20.8%. However, the different treatment setting with different OAR constraints makes it hard to extrapolate these results to our study. Currently, Zilli et al. are investigating the delivery of a single 19 Gy dose to the whole prostate gland and two-thirds of the seminal vesicles with urethra sparing (down to 17 Gy) in a primary setting [17], [18]. For bladder and rectum constraints, they use V20Gy < 1 cm^3^. Again, because of the different setting and constraints, the results of this study are not directly comparable to our results.

OAR constraints were met for all MR-Linac plans. We found a higher median urethral and rectal dose with the MR-Linac plans compared to FS-HDR-BT. This can be explained by the more gradual dose fall-off with SBRT and the larger PTV. Besides a higher rectum D1cm^3^, the mean dose to the rectum is also likely to increase. However, limited data is available on toxicity in patients treated with focal salvage radiotherapy. In our FS-HDR-BT group, grade 2 and higher gastro-intestinal (GI) toxicity was extremely low, with no grade 3 GI toxicity so far and only 4% new-onset grade 2 GI toxicity, as reported by Van Son et al. [19]. Although rectal dose is slightly higher with SBRT, it is still below the constraint and therefore low GI toxicity is expected. Bladder D1cm^3^ and D2cm^3^ were comparable for FS-HDR-BT and MR-Linac. This is probably caused by the more cranial position of the bladder with respect to the prostate and therefore this organ is easier to avoid with SBRT than the rectum. Again, the differences were quite small and probably clinically irrelevant. Nevertheless, prospective studies should assess this.

As expected, larger volumes receiving 150% or 200% of the prescribed dose were reached with FS-HDR-BT. A means to increase the volume receiving high dose levels using a MR-Linac might be to include an additional – higher – dose prescription to the GTV. A higher dose to the tumor potentially leads to longer biochemical progression-free survival (BPFS). However, the exact relationship between these high-dose volumes and BPFS in this patient group has to be established. Also, especially in this patient group with recurrent prostate cancer, radioresistance can play a role in oncologic outcomes of the treatment. Overall BPFS ranges between 47% and 54% at 5-year follow-up and a recent update by Van Son et al. showed that 73% of the intraprostatic recurrences after FS-HDR-BT occurred in-field [19], [20]. Accordingly, this may imply that treatment of these tumors might benefit from an increased dose. Dose escalation and/or fractionation could improve oncologic outcomes, but may go hand-in-hand with increased acute toxicity as suggested by Murgic et al. [21]. If future study results demonstrate improved oncologic outcomes by applying e.g. two or three fractions, the MR-Linac is a more attractive treatment modality due to both the non-invasiveness and easier logistics.

Stratifying the target dose by tumor location showed areas with higher and lower median D95%. However, the low numbers complicate drawing conclusions about these observations. Obviously, reaching target coverage strongly depends on the anatomy and therefore a decision should be made for each patient individually.

This study has several strengths. We included 30 patients with various tumor locations and sizes, thereby showing the feasibility in a non-selected patient group comparable to the target population. Secondly, from a FS-HDR-BT perspective, we used clinically delivered plans, which reflect the real capabilities of the FS-HDR-BT treatment modality.

Conversely, a limitation of this study is the fact that the MR-Linac plans are optimal plans based on several assumptions. Firstly, we have used a 1 mm PTV margin. As discussed in the materials and methods section, this 1 mm margin has to account for additional uncertainty due to intrafraction prostatic motion and geometrical (in)accuracy that are introduced with the use of a MR-Linac system compared to FS-HDR-BT. Based on the mentioned prostate tracking algorithm and adaptive treatment planning systems that have been developed at our department, this 1 mm margin seems to be achievable [9], [11], [13], [14]. Since interobserver contouring variability of the target is present in FS-HDR-BT as well, we do not expect this to lead to additional inaccuracy with MR-Linac treatments. Also, we believe that the full potential of MR-Linac systems lies in achieving these very small PTV margins that are not possible with conventional systems. Based on our prostate motion analyses, 3 to 4 mm margins are sufficient for current prostate cancer treatment without intra-fraction adaptation [12]. Enlarging the PTV margin in our study to i.e. 3 or 5 mm would thus not make full use of the MR-Linac system’s capabilities that distinguish it from conventional cone-beam CT linear accelerators. Still, this will remain hypothetical until it is technically possible to combine all the aforementioned developments into a clinically released system. Another limitation is the fact that we only had full field-of-view CT scans available for MR-Linac treatment planning. The registration between the intraoperative MRI and the CT scan and the contour propagation could lead to differences in the exact dose distributions. However, these potential differences were assumed to be minor and have no impact on the planning comparison study. Thirdly, we used FS-HDR-BT OAR constraints for our evaluation. We assessed the highest dose to small volumes (1 cm^3^) for both the rectum and bladder. Contrary to FS-HDR-BT, mean dose to OAR is generally higher with external beam radiotherapy due to the more gradual dose fall-off. However, delivery of a single 19 Gy dose to a focal recurrent tumor using a volumetric modulated arch therapy (VMAT)-like technique resembles SBRT. Several studies on ultra-hypofractionated and single session radiotherapy for prostate cancer use only high-dose to low-volume constraints, thereby showing low toxicity rates [18], [22], [23], [24]. Accordingly, we argue that D1cm^3^ is a sensible and important parameter allowing for comparison between the two treatment modalities. Fourthly, due to the small stratified sample sizes, we were unable to draw any definitive conclusions from the dosimetric analysis per tumor location. Lastly, by using the intra-operative delineations from the FS-HDR-BT treatment, we took into account the effect of volume changes (edema) that might have occurred during brachytherapy due to the insertion of catheters. While no volume changes will occur with MR-Linac treatment, we used the same delineations for MR-Linac treatment planning. Therefore, the volumes might not be completely representative for MR-Linac treatment. However, for a fair comparison of the dose distributions, no adaptations were made to the delineations to include identical target volumes for both treatment plans.

In conclusion, we showed that for the majority of the patients treated with FS-HDR-BT, we could create an acceptable and comparable MR-Linac plan. This demonstrated the feasibility of SBRT treatment planning for radiorecurrent prostate cancer using a single 19 Gy dose on a 1.5 Tesla MR-Linac system, while respecting FS-HDR-BT OAR constraints. Hence, MR-Linac systems may provide a non-invasive alternative to FS-HDR-BT. Research should be conducted on the necessity of the larger high-dose volumes achieved in FS-HDR-BT, which may favor oncological outcomes in the long-term. Before clinical application of salvage MR-Linac treatment, real-time intra-fraction adaptation and dose calculation should be technically enabled, and clinical feasibility has to be tested in early phase clinical trials.

## 5 Role of the funding source

This research has been partly funded by 10.13039/501100001826ZonMw (The Netherlands, IMDI project, grant 104006004), the LSH-TKI Foundation (The Netherlands, grant 104006004), and the 10.13039/501100004622Dutch Cancer Society (The Netherlands, grant 10392). The funding sources had no involvement in the design of the study, the collection, analysis, and interpretation of the data, nor in the writing and decision to submit the article for publication.

## Declaration of Competing Interest

The authors declare that they have no known competing financial interests or personal relationships that could have appeared to influence the work reported in this paper.
