# Supplementary material for: Focal salvage treatment for radiorecurrent prostate cancer: A magnetic resonance-guided stereotactic body radiotherapy versus high-dose-rate brachytherapy planning study
Source: Phys Imaging Radiat Oncol. 2020 Aug 7;15:60–5. doi: 10.1016/j.phro.2020.07.006 (PMC7807590; doi:10.1016/j.phro.2020.07.006)
Supplement: Supplementary data 1 [file mmc1.docx]

Supplementary Material

**Table S1** Baseline tumor characteristics.

| Baseline characteristic | Number of patients  (total N = 30) |
| --- | --- |
| Local recurrence – side of prostate  Left Right  Both | 8 (27%)  14 (47%)  8 (27%) |
| Local recurrence – location with respect to organs at risk  Peripheral, not near any of the OARs  Central, adjacent to urethra  Peripheral/lateral, between rectum and urethra  Central, between rectum and urethra  Central, Between/adjacent to bladder and urethra  Peripheral, adjacent to rectum (not near urethra)  Seminal vesicles, adjacent to rectum and/or bladder  Base, adjacent to bladder and rectum, no seminal vesicle involvement | 5 (17%)  3 (10 %)  3 (10%)  4 (13%)  3 (10%)  4 (13%)  5 (17%)  3 (10%) |
| Mean GTV [range] | 4.9 cm^3^ [0.7-17.8 cm^3^] |
| Mean CTV [range] | 11.9 cm^3^ [2.9-24.7 cm^3^] |
